# Intra-/Interobserver Agreement of Enhancement Pattern for Pancreatic Head Lesions Using Contrast-Enhanced Ultrasound: According to EFSUMB Guidelines

**DOI:** 10.3390/diagnostics2020002

**Published:** 2012-04-20

**Authors:** Hanne Sønder Grossjohann, Caroline Ewertsen, Lars Bo Svendsen, Michael Bachmann Nielsen

**Affiliations:** Department of Radiology, Section of Ultrasound X4123, Copenhagen University Hospital, Rigshospitalet, Blegdamsvej 9, Copenhagen DK 2100, Denmark; E-Mails: hannesonder@dadlnet.dk (H.S.G); caroline.ewertsen@dadlnet.dk (C.E); larsbo.svendsen@dadlnet.dk (L.B.S); mbn@dadlnet.dk (M.B.N)

**Keywords:** contrast-enhanced ultrasound (CEUS), pancreatic head lesions, intra-/interobserver agreement

## Abstract

*Objectiv*e: To evaluate the intra-/interobserver agreement of the visual interpretation of contrast-enhanced ultrasound (CEUS) of pancreatic head lesions and its concordance with the histological test results. *Material and Methods*: Two observers (A + B) evaluated by simple visual interpretation 40 consecutive CEUS examinations of pancreatic head lesions and one of the observers evaluated the examinations twice (A1 + A2). The examinations were evaluated according to the criteria outlined in EFSUMB guidelines. The two experienced observers were blinded to histological evidence and clinical information of tumor type and to each other’s results. *Results*: The kappa value for the intraobserver evaluation between observer A1 and A2 was 0.89, equating to almost perfect agreement. The kappa value for the interobserver evaluation between observer A1 and B was 0.76 and between observer A2 and B it was 0.75, both equating to substantial agreement. Evaluation of the visual interpretation compared to the histological test result showed a positive predictive value for A1, A2 and B *versus* biopsy of 97%, 94% and 90% respectively and an accuracy of 83%, 88% and 73% respectively. *Conclusions*: Visual interpretation for assessment of contrast enhancement of pancreatic head lesions seemed to be an accurate method with reproducible results and good concordance with the histological test results.

## 1. Introduction

Agreement of interpretation of diagnostic tests is an essential issue because the diagnostic test will be of little use if the observers do not agree [1]. The use of ultrasound contrast agents has been increasingly accepted in clinical use for diagnostic imaging firstly of the liver but recently in several organs including the pancreas [2,3,4,5,6,7,8,9,10,11,12,13,14]. In the newest edition of European Federation of Societies for Ultrasound in Medicine and Biology (EFSUMB) guidelines and good clinical practice recommendations for contrast-enhanced ultrasound (CEUS), there is a section concerning CEUS of pancreatic tumors [15]. It is suggested that CEUS can be used to characterize ultrasound-detected tumors; however this indication has not yet obtained regulatory approval. All the referred manuscripts for this chapter in the EFSUMB guidelines are based on visual evaluation.

The aim of this study was to test the intra-/interobserver agreement of visual interpretation and its concordance with the histological test results. We wished to test the visual interpretation because it is the most frequently used method to day [15].

## 2. Methods

We evaluated 40 consecutive CEUS examinations of pancreatic head lesions (19 women and 21 men; age range, 42–83 years; mean age, 66 years) obtained over a period of two years (December 2005−December 2007). Thirty-five of the patients had adenocarcinoma and five had chronic pancreatitis. The mean tumor size was 2.7 cm (range 1–8 cm, SD 1.4). Only the pancreatic head tumors were included in the study, and not all pancreatic tumors, in order to have the most uniform data material for the analysis.****The Local Ethics Committee approved the study and all patients gave informed consent to participate. All patients were referred to our hospital, Center for Pancreas Cancer from other hospitals because of radiological findings of pancreatic head lesions and suspicion of pancreatic cancer. They were included in a diagnostic research protocol where the pancreatic head lesions were examined by ultrasound, CEUS and 64-slice-CT in the mentioned sequence. All the examinations were made on the same day with a short time span of up to two hours in between the ultrasound examination and the 64-slice-CT examination. The patients were on an outpatient basis and were asked only to eat a light meal at least four hours before the first examination. The ultrasound equipment used for the examinations was a GE Logiq 9 (GE Healthcare, Milwaukee, WI, USA) with a 2 to 4 MHz curved array wideband transducer. The transabdominal scan was supported using color Doppler, tissue harmonic, crossbeam, and speckle reduction. The CEUS was performed with a mechanical index set as default at 0.12 and the acoustic output was 100%. The CEUS examination was supported using dual screen (a setting with the B-mode image and the contrast image shown simultaneously) and all examinations were stored as 40 seconds long cineloop sequences covering a period of two minutes (arterial and venous phases). Before scanning with contrast agent the pancreatic lesion was fixed in the optimal position and the transducer was kept in that position throughout the contrast examination. The contrast agent SonoVue (Bracco, Milan, Italy) was manually injected as a bolus injection of 2.4 mL through a 20-gauge cannula placed in an antecubital vein. After each bolus the cannula was flushed with 5 mL saline solution.

The enhancement patterns of the pancreatic lesions were graded visually into three categories: low or less contrast enhancement (hypoenhancing), homogenous enhancement (isoenhancing) and marked enhancement (hyperenhancing). Hypoenhancement of a lesion was interpreted as the lesion being an adenocarcinoma, isoenhancement as the lesion being a pseudo tumor originating from chronic pancreatitis and hyperenhancement as the lesion being either a metastasis or a neuroendocrine tumor. This method has been used by all referred manuscripts in the EFSUMB guidelines [15] and is based on knowledge of the vascular construction of the different lesions. All the cases were histological or cytological verified and/or subsequently followed up.

The agreement study was first thought to be an intraobserver study only (A1 and A2). But since we had the opportunity and the data material saved on a computer we found that involvement of a second observer (B) would strengthen the study in the form of an interobserver evaluation. The second observer’s (B) evaluation and the second evaluation of the first observer (A2) were made six months after the image footage. This time span should prevent or minimize any recollection of the examinations for the first observer (A1). The second observer had never seen any of the examinations before she evaluated them. Theoretically the enhancement pattern should appear the same immediately after the footage and six months or anytime later, ideally for any observer. It should always, at any time after the footage be possible to visually re-evaluate a CEUS examination and ideally come to the same conclusion about the enhancement pattern over and over again. All examinations were evaluated by looking at the stored cineloop sequences on a computer workstation. The enhancement pattern of each pancreatic lesion was evaluated by looking at it during the two minutes of footage and afterwards a decision was made whether the lesion appeared hypoenhancing, isoenhancing or hyperenhancing. Afterwards the results were statistically compared. The two experienced observers were blinded to histological evidence of tumor type, to clinical information and to each other’s results.

We used kappa statistics for the analysis of the intra-/interobserver results. Kappa is a measure of the difference between an observed agreement and an expected agreement. It is standardized to lie on a −1 to 1 scale, where 1 is perfect agreement, 0 is exactly what would be expected by chance and negative values indicate agreement less than chance [1,16]. The values are translated into a categorical scale (Table 1):

**Table 1:** Interpretation of Kappa.

Kappa	Agreement
<0	Less than change agreement
0.01–0.20	Slight agreement
0.21–0.40	Fair agreement
0.41–0.60	Moderate agreement
0.61–0.80	Substantial agreement
0.81–0.99	Almost perfect agreement

When comparing the visual interpretation to the histological test results we used binary classification tests. Sensitivity, specificity, positive predictive value (PPV), negative predictive value (NPV) and accuracy were calculated.

**Table 2:** Distribution of the observations concerning isoenhancement and hypoenhancement.

	A1			
A2		Hypo-enhanced	Iso-enhanced	Total
	Hypo-enhanced	30	4	34
	Iso-enhanced	0	6	6
	Total	30	10	40

	A1			
B		Hypo-enhanced	Iso-enhanced	Total
	Hypo-enhanced	25	5	30
	Iso-enhanced	4	6	10
	Total	29	11	40

	A2			
B		Hypo-enhanced	Iso-enhanced	Total
	Hypo-enhanced	27	2	29
	Iso-enhanced	7	4	11
	Total	34	6	40

**Table 3:** Kappa values for the distribution pattern of the observer’s observations concerning isoenhancement and hypoenhancement.

	Numerical value	Categorical value
A1 and A2	K = (0.9 − 0.11)/(1 − 0.11) = 0.89	Almost perfect agreement
A1 and B	K = (0.78 − 0.1)/(1 − 0.1) = 0.76	Substantial agreement
A2 and B	K = (0.78 − 0.12)/(1 − 0.12) = 0.7	Substantial agreement

**Figure 1:**
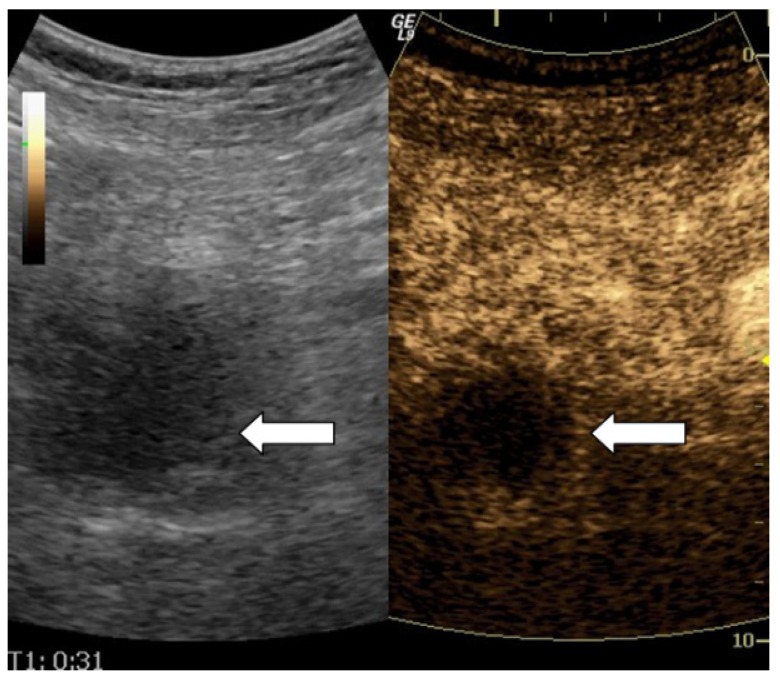
Dual scan image of a pancreatic lesion. B-mode image to the left and contrast image to the right. All observers agreed that this lesion was hypoenhanced. The radiological diagnosis was adenocarcinoma and this was in concordance with histology.

## 3. Results

**Figure 2:**
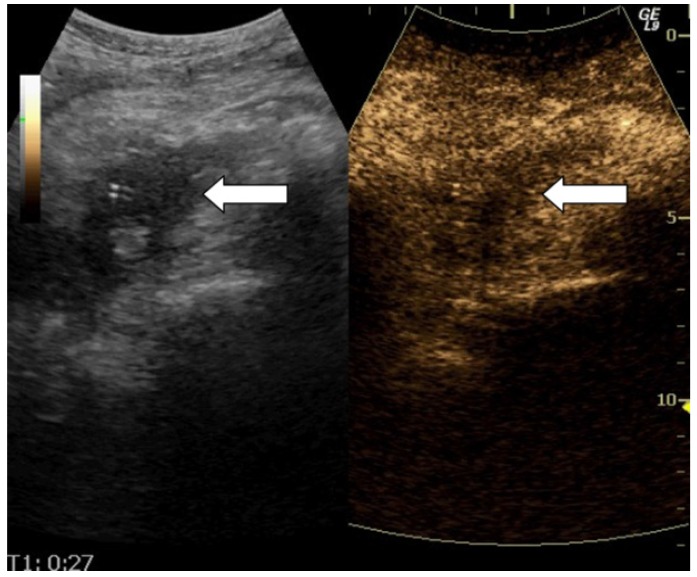
Dual scan image of a pancreatic lesion. B-mode image to the left and contrast image to the right. Observer A1 and B judged isoenhancement and observer A2 judged hypoenhancement. The histological diagnosis was adenocarcinoma.

None of the observers judged the enhancement of any of the pancreatic lesions to be hyperenhanced. Table 2 shows the distribution of the observer’s observations concerning isoenhancement and hypoenhancement. The observed agreement is 90% (36/40) for A1 and A2, 78% (31/40) for A1 and B, and 78% (31/40) for A2 and B. Table 3 shows the kappa values. Table 4 shows the distribution of the observations concerning the visual interpretation of the contrast-enhancement compared to the histological test results. The sensitivity for A1, A2 and B *versus* biopsy was 83% (29/35), 91% (32/35) and 77% (27/35) respectively, the specificity was 80% (4/5), 60% (3/5) and 40% (2/5) respectively, the positive predictive value was 97% (29/30), 94% (32/34) and 90% (27/30) respectively, the negative predictive value was 40% (4/10), 50% (3/6) and 20% (2/10) respectively and the accuracy was 83% (33/40), 88% (35/40) and 73% (29/40) respectively. Table 5 shows the evaluation of the visual interpretation of the contrast-enhancement compared to biopsy as gold standard. Figure 1)and Figure 2 both illustrate two of our examinations of contrast enhancement of pancreatic lesions.

**Table 4:** Distribution of the observations concerning the visual interpretation of the contrast-enhancement compared to biopsy.

	Biopsy			
A1		Adenocarcinoma	Pancreatitis	Total
	Hypo-enhanced	29	1	30
	Iso-enhanced	6	4	10
	**Total**	35	5	40

	Biopsy			
A2		Adenocarcinoma	Pancreatitis	Total
	Hypo-enhanced	32	2	34
	Iso-enhanced	3	3	6
	**Total**	35	5	40

	Biopsy			
B		Adenocarcinoma	Pancreatitis	Total
	Hypo-enhanced	27	3	30
	Iso-enhanced	8	2	10
	**Total**	35	5	40

**Table 5:** Evaluation of the visual interpretation of the contrast-enhancement compared to biopsy as gold standard. Positive predictive value = PPV, negative predictive value = NPV.

Group	Sensitivity (95% CI)	Specificity (95% CI)	PPV (95% CI)	NPV (95% CI)	Accuracy (95% CI)
A1 *versus* **Biopsy	83% (71–95)	80% (68–92)	97% (92–102)	40% (25–55)	83% (71–95)
A2 *versus* **Biopsy	91% (82–100)	60% (45–75)	94% (87–101)	50% (35–66)	88% (78–98)
B *versus* **Biopsy	77% (64–90)	40% (25–55)	90% (81–99)	20% (8–32)	73% (59–87)

## 4. Discussion and Conclusions

In the present study our intra-/interobserver agreements of the visual interpretation of enhancement pattern of pancreatic lesions using CEUS showed substantial or almost perfect agreement with kappa values between 0.75 and 0.89. Likewise the visual interpretation of enhancement pattern seemed to be in concordance with the histological test results with overall accuracies between 73% and 83%.

The EFSUMB guidelines report that hypoenhancement of a pancreatic lesion compared to the adjacent pancreatic tissue typically appears in 88–93% of the cases [15]. In some cases however, isoenhancement also appears in an adenocarcinoma. This could be because of the presence of small vessels around and/or in the tumor and this gives the tumor a mesh or spotty signal, which results in a heterogeneous enhancement in the hypoenhanced pancreatic tumor [13]. Another case of assessed inconsistency between hypoenhancement and isoenhancement could be linked up with the fact that not all pancreatic lesions are cytologically unambiguous. In several cases we experienced that the primary cytological answer sounded was; “suspicion of malignancy” or “atypical cells”. However, in these cases the final histological answer always sounded was; adenocarcinoma. The Kersting *et al*. group mentions that inflammatory tissue is found often in the vicinity of a pancreatic adenocarcinoma and that this potentially can lead to misdiagnoses because of the appearance of mixed signals at CEUS [17]. The D’Onofrio *et al*. group found that the more markedly hypovascular the pancreatic masses appeared at CEUS the greater the aggressiveness of the tumor and the worse the prognosis [18]. This corresponds to our observations that the more markedly hypovascular the pancreatic lesion appeared the better the intra-/interobserver agreement and the better the correspondence to the histological test result. The Xu *et al*. group and the Bertolotto *et al*. group proved that the visual interpretation of CEUS examinations on different organs yield a high intra-/interobserver agreement [19,20].

A limitation in this study could be that the patient data used for this examination was selected. All patients included in the study were referred to our hospital for operation because of suspicion of pancreatic cancer and they were considered resectable on the primary radiological imaging material. This referral practice may differ from that at other institutions and it is possible that knowledge about the primary diagnosis of these patients may influence the observers to interpret the CEUS images in a malignant direction. Another limitation of the study is that the population of patients with potentially resectable pancreatic tumors is rather small. That is why our study is based on a relatively small amount of sample data, which of cause is a statistical shortcoming but inevitable. In conclusion visual interpretation for assessment of contrast enhancement of pancreatic head lesions seemed to be an accurate method with reproducible results and good concordance with the histological test results.
